# Explore the interaction between root metabolism and rhizosphere microbiota during the growth of *Angelica sinensis*

**DOI:** 10.3389/fpls.2022.1005711

**Published:** 2022-11-07

**Authors:** Jing-Mei Chen, Wei-Meng Feng, Hui Yan, Pei Liu, Gui-Sheng Zhou, Sheng Guo, Guang Yu, Jin-Ao Duan

**Affiliations:** Jiangsu Collaborative Innovation Center of Chinese Medicinal Resources Industrialization, National and Local Collaborative Engineering Center of Chinese Medicinal Resources Industrialization and Formulae Innovative Medicine, Nanjing University of Chinese Medicine, Nanjing, China

**Keywords:** *Angelica sinensis*, rhizosphere microbiota, metabolomics, plant development stage, correlation, quality

## Abstract

*Angelica sinensis* is a medicinal plant widely used to treat multiple diseases in Asia and Europe, which contains numerous active components with therapeutic value. The interaction between root and rhizosphere microorganisms is crucial for the growth and quality formation of medicinal plants. But the micro-plant-metabolite regulation patterns for *A. sinensis* remain largely undetermined. Here, we collected roots and rhizosphere soils from *A. sinensis* in seedling stage (M) and picking stage (G), respectively cultivated for one year and two years, generated metabolite for roots, microbiota data for rhizospheres, and conducted a comprehensive analysis. Changes in metabolic and microbial communities of *A.sinensis* over growth were distinct. The composition of rhizosphere microbes in G was dominated by proteobacteria, which had a strong correlation with the synthesis of organic acids, while in M was dominated by Actinobacteria, which had a strong correlation with the synthesis of phthalide and other organoheterocyclic compounds, flavonoids, amines, and fatty acid. Additionally, co-occurrence network analysis identified that *Arthrobacter *was found to be strongly correlated with the accumulation of senkyunolide A and n-butylidenephthalide. *JGI 0001001.H03 *was found to be strongly correlated with the accumulation of chlorogenic acid. Based on rhizosphere microorganisms, this study investigated the correlation between root metabolism and rhizosphere microbiota of *A. sinensis* at different growth stages in traditional geoherb region, which could provide references for exploring the quality formation mechanism of *A. sinensis* in the future.

## Introduction

*Angelica sinensis* (Oliv.) Diels (Umbelliferae family), known as Dang Gui (in Chinese), is a traditional medicinal and edible plant which was recommended as a first-line treatment for gynecological diseases and is widely used in Asia and Europe. *A. sinensis* is mainly used in treating female menstrual disorders and amenorrhea, as well as invigorating and replenishing blood, and lubricating the intestines (Hook, 2014; Wei et al., 2016). Numerous pharmacological effects of *A. sinensis* include enhanced immune function, anti-arrhythmic, heart protection, anti-atherosclerotic, and inhibited platelet aggregation and smooth muscle inflammation to prevent myocardial infraction events (Hu et al., 1991; Liu et al., 2001; Zhou et al., 2015; Yao et al., 2015). Diverse habitats and climates have shaped the qualities and efficacy of medicinal plants. In China, it is generally believed that Geo-authentic Chinese medicinal materials are selected by long-term clinical application which considered with satisfactory clinical efficacy and stable quality and produced in specific ecological environment. A Chinese group standard, *Daodi Chinese Medicinal Material – MinDanggui*, defines the geoherb region of *A. sinensis* explicitly. Standard states that the geoherb region of *A. sinensis* are Min County and its surrounding areas, such as Tanchang, Zhang, Weiyuan, Zhuoni, Lintan Counties in Gansu province, which are located in the eastern branch of Minshan Mountains in southern Gansu province and the transitional zone where loess Plateau in Longzhong and the Qinghai – Tibet Plateau meet (Huang, Guo, & Zhan, 2020). *A. sinensis* is a typical ecological dominant medicinal plant, and ecological environment is an essential factor affecting the quality of traditional Chinese medicine (TCM). Min County and its surrounding areas are located between 33°46′~35°07′ N, 103°14′~104°59′ E, the altitude ranges from 2040 m to 3747 m, with cool humid climate, moderate rainfall, rich brown soil and black soil, which are favorable to the growth of *A. sinensis* and are the main areas for the production of *Angelica sinensis* (Zhang et al., 2016). In 2014, the *Radix Angelica sinensis* planting system in Min County of China was selected as the second batch of China-NIAHS because of its unique cultivation conditions, traditional cultivation techniques, and processing techniques, as well as the indigenous microbial flora of Min County, which plays a vital role in shaping the quality of *A. sinensis.*

Secondary compounds in plants (PSCs) are crucial messengers in plant ecology through chemical interactions with plant hosts and the outside world. For example, they can attract herbivorous predators and pollinators to help spread seeds by releasing secondary metabolites. Plant chemical barriers are also formed to prevent invasion by pathogens and herbivorous predators. In turn, pathogen and predator pressures, microbes, and microclimates all influence the levels and types of secondary metabolites that plants synthesize and release (Ehlers et al., 2020). Studies have shown that phthalides, organic acids, and polysaccharides are the primary secondary metabolites in determining the biological activities and pharmacological properties of *A. sinensis* (Wei et al., 2016). The root microbial community could affect the growth and yield of *A. sinensis*. Studies have shown that Bacillus isolated from the rhizosphere can promote biomass accumulation and plant growth, and can increase the contents of butylidenephthalide (36 ~ 415%) of *A. sinensis* (Feng et al., 2020). Nonetheless, little is known about the mechanisms of the accumulation of *A. sinensis* metabolites and the microbial communities. Soil microbial diversity is closely related to ecosystem functions such as nutrient decomposition and recycling, and positively related to plant productivity in the earth’s microbiome (Delgado-Baquerizo et al., 2020). Plant rhizosphere is the most abundant part of microbial activity. Plants selectively assemble specialized functional rhizosphere microbiota from bulk soil for plant fitness. On the other hand, many rhizosphere microorganisms benefit plants by assisting with acquiring supplements from soil and suppressing pathogen invasion (Bulgarelli et al., 2013; Leach et al., 2017; Ling et al., 2022). The variety and composition of rhizosphere bacterial communities result from a combination of plant species and soil properties (Ling et al., 2022). However, these plant microbes are not constant; they vary with environmental stimuli, including abiotic stress and biotic factors. The microbiota structure is impacted by the physical and chemical aspects of the environment (Pang et al., 2021). It has been determined that the rhizosphere microbiota is a highly modular but unstable network system by the effects of plant hosts and other microbes. This characteristic reflects the interaction between microorganisms and their adaption to dynamic conditions (Ling et al., 2022). Studies have shown that root metabolism and microbiota are sensitive to and driven by changes in plant growth stages. Results showed a correlation between stable and dynamic root microbial taxa and root lipids during plant growth (Bourceret et al., 2022). Plants can influence their microbiome by secreting a variety of metabolites, which in turn can influence the metabolome of host plants (Pang et al., 2021). The chemical assemblage of microbial communities and the composition of root exudates vary in the stages of plant growth. The underlying mechanism may be related to the substrates preference of microorganisms (Zhalnina et al., 2018). On the other hand, the same plant growing in different habitats may affect the type and content of plant secondary metabolites, and these differences may be closely related to indigenous microbes (Köberl et al., 2013). *A. sinensis* is an herbaceous perennial plant. In cultivation, it has a three-year growth cycle. Traditionally, seedlings are fostered in uncultivated alpine meadows in the first year, transplanting the seedlings, harvesting the fleshy roots in the second year, and collecting the seeds in the third year (An et al., 2020). The cultivation pattern of transplanting determined that *A. sinensis* had to adapt to different habitats and corresponding microbial environments during its growth. Studies of specific ecosystems observed that alpha-diversity in rhizosphere of cultivated land continued to decline (-0.8% ~ -9.3%), while in grassland and forest ecosystems, there were no significant differences in Faith’s phylogenetic diversity and species richness between the rhizosphere and bulk soil (Ling et al., 2022). Therefore, the microbial environment in different habitats has different shaping effects on the quality of medicinal plants. Root length, soil pH, climate temperature, rainfall, root diameter, and plant weight are also considerable factors affecting the composition of *A. sinensis* rhizosphere microbial community (An et al., 2020).

Research on rhizosphere microbiome for sustainable ecosystem development has focused on identifying the core of plant microbiota and clarifying the functional mechanisms that regulating plant-microbiome interactions. (Busby et al., 2017; de Vries et al., 2020). Here, we collected 14 batches of annual seedlings from the traditional geoherb region of *A. sinensis* and planted them in the same experimental field in Min county, Gansu province. The same measures of cultivation and management were carried out during the planting period, and it was allowed to grow for 180 days before harvest. Seedlings and mature roots of *A. sinensis* were measured the root metabolites and medicinal components using UPLC-QTOF-MS/MS and performed 16S rRNA sequencing on rhizosphere samples. We generated metabolites profiles, medicinal components and microbial community composition for seedlings (M) and mature roots (G) of *A. sinensis*, profiled the differential metabolites and medicinal components of *A. sinensis* over growth, deciphered the compositional characteristics of microbes colonizing *A. sinensis* roots in different growth stage, and characterized the dynamic regulations between the accumulation of secondary metabolites and rhizosphere microbial community. We focused the following questions during the *A. sinensis* transplanting cultivation model: (1) Does the synthesis of secondary metabolites and the contents of principal bioactive constituents of *A. sinensis* change at seedling stage and picking stage? (2) The assembly of rhizosphere microbial community changes with the different growth stage. How does the rhizosphere microbial community of *A. sinensis* assemble over growth? (3) Are there any correlations between the accumulation of secondary metabolites and the change of rhizosphere microbial community?

## Materials and methods

### Sample collection

Collection of 14 batches of seedlings of annual *A. sinensis* in Min County, Tanchang County, Zhang County, Lintan County and Zhuoni County in Gansu Province, China. The seedlings were planted in Min County Medicinal Plants Growing Technology Extension Centre (34°22′30” N, 104°53′20” E; black soil, pH ≈ 8.0) with same measures of cultivation and management during the planting period, and it was cultivated to grow for 180 days then harvest. Three samples for each batch were randomly selected, each sample was comprised of 5 healthy plants, and 42 samples were taken for each growth stage. The seedlings and mature roots were both authenticated by Dr. Hui Yan. Carefully, uproot fresh plants and shake loose bulk soil that clings to the roots. Remove the rhizosphere soil which tightly attached to roots with a sterile brush. Fresh plants and soil samples were stored at -80 °C refrigerator for analyses.

### Soil DNA extraction and sequencing

According to manufacturer’s approach, total DNA was extracted from per sample using the HiPure Soil DNA Kits (Magen, Guangzhou, China). The bacterial sequence was amplified with the primers 806R (5’-GGACTACHVGGGTATCTAAT-3’) and 341F (5’-CCTACGGGNGGCWGCAG-3’). PCR reactions were performed in triplicate in 50 μL mixture containing 3 μL of 25 mM MgSO_4_, 5 μL of 2 mM dNTPs, 5 μL of 10 × KOD Buffer, 1.5 μL of each primer (10 μM), 100 ng of template DNA, and 1μL of KOD Polymerase. The bacterial V3-V4 hypervariable region of 16S rRNA was amplified by PCR (94°C for 2 min, followed by 30 cycles at 98°C for 10 s, 62-66°C for 30 s, and 68°C for 30 s, and a final extension at 68°C for 5 min) using primers. Related PCR reagents were from TOYOBO, Japan. According to the manufacturer, the PCR amplicons were extracted from 2% agarose gels and purified using the AMPure XP Beads (Beckman Agencourt, USA), and quantified using ABI StepOnePlus Real-Time System (Life Technologies, Foster City, USA). Purified amplicons were pooled in equimolar and paried-end sequenced (PE250) on an Illumina platform in accordance with the standard protocols.

### Bioinformation analysis

FASTP (Chen et al., 2018) (version 0.18.0) mainly used for the quality control of the raw reads which containing less than 50% of bases with quality (Q-value)>20 and more than 10% of unknown nucleotides (N). The splicing was done by FLASH (Magoc and Salzberg, 2011) (version 1.2.11). The clean tags were grouped into operational taxonomic units (ASVs) by UPARSE (Edgar, 2013) (version 9.2.64) according to the similarity ≥ 97%. The representative OUT sequences was analyzed by RDP classifier (Wang et al., 2007) (version 2.2) based on SILVA (Pruesse et al., 2007) database (version 132), were classified into organisms according to the confidence threshold of 0.8. Diversity indices of data were performed in QIIME (version 1.9.1) and R packages (version 2.5.3). Statistic analysis of Anosim test, Tukey’s HSD test, Welch’s t-test, Kruskal-Wallis H test, Wilcoxon rank test and Adonis was calculated in R project Vegan package (version 2.5.3).

### Medicinal components and metabolite analysis

The ACQUITY™ UPLC system was used to perform the medicinal components of *A. sinensis* with an A Thermo Syncronis C_18_ (2.1 mm × 100 mm, 1.7 μm) column. The 0.1% formic acid in chromatographic (A) and acetonitrile (B) were used as mobile phase. The gradient elution were as follow: 0 ~ 2 min, 5% ~ 10% B; 2 ~ 10 min, 10% ~ 40% B; 10 ~ 13 min, 40% ~ 40% B; 13 ~ 19 min, 40% ~ 50.8% B; 19 ~ 23 min, 50.8% ~ 90% B; 23 ~ 24 min, 90% ~ 90% B; 24 ~ 24.5 min, 90% ~ 5% B; 24.5 ~ 26 min, 5% ~ 5% B The detection wavelength are 260 nm, 280 nm and 320 nm. The medicinal components (ferulic acid, chlorogenic acid, Z-ligustilide, senkyunolide A, senkyunolide H, senkyunolide I, n-butylphthalide, coniferyl ferulate) were measured by comparing with the calibration curves. The 8 reference compounds bought from National Institutes for Food and Drug Control (China), Shanghai Macklin Biochemical Co., Ltd (China) and Nanjing Jin Yibai Biological Technology Co., Ltd (China) respectively. The Waters ACQUITY™ Synapt Q-TOF mass spectrometer equipped with an electrospray ionization (ESI) source. ESI-MS spectra was generated in both positive (ESI^+^) and negative (ESI^-^) ion modes through scanning from 100-1000 Da. The flow rate was 0.4 mL·min^-1^, column temperature was kept at 35°C, with 2 μL injection volume. The optimized conditions include: capillary voltage 3 kV for both positive ion mode and negative ion mode; source temperature 120°C; desolvation gas temperature 350°C; desolvation gas flow 600 L/h; sample cone voltage 30 V; cone gas flow 50 L/h; extraction cone voltage 2.0 V; secondary collision energy 25 ~ 45 V. Quantitative mass spectrometric analysis was performed using Xevo Triple Quadrupole MS (Waters Corp., Milford, MA USA) equipped with electrospray. ESI-MS spectra were obtained by using MRM mode.

### Statistical analyses

Using IBM SPSS Statistics 19 and R (4.2.0) to perform all statistical analyses. The mean and standard deviation were calculated by multiple comparison analysis and analysis of variance (ANOVA) for statistical tests. The univariate approach depends on t-tests (or their nonparametric alternatives). Spearman correlation coefficients were calculated using R v 4.2.0. RStudio v 2022.02.1 and SIMICA v14.1 were employed to perform PCA and OPLS-DA. Differences between groups were considered significant when *p* < 0.05. TBtools v 1.09876 (Chen et al., 2020) was employed to perform heatmaps and hierarchical clustering. Statistical analysis of rhizosphere microorganisms taxonomic differences between groups using STAMP v 2.1.3. Cytoscape v3.9.1 and MetScape v3.1.3 were employed to visualize the co-occurrence networks of microbiota and metabolome data. Results were built and optimized using OriginPro 2021.

## Result

### Bacterial alpha-diversity and beta-diversity

**Figure 1:**
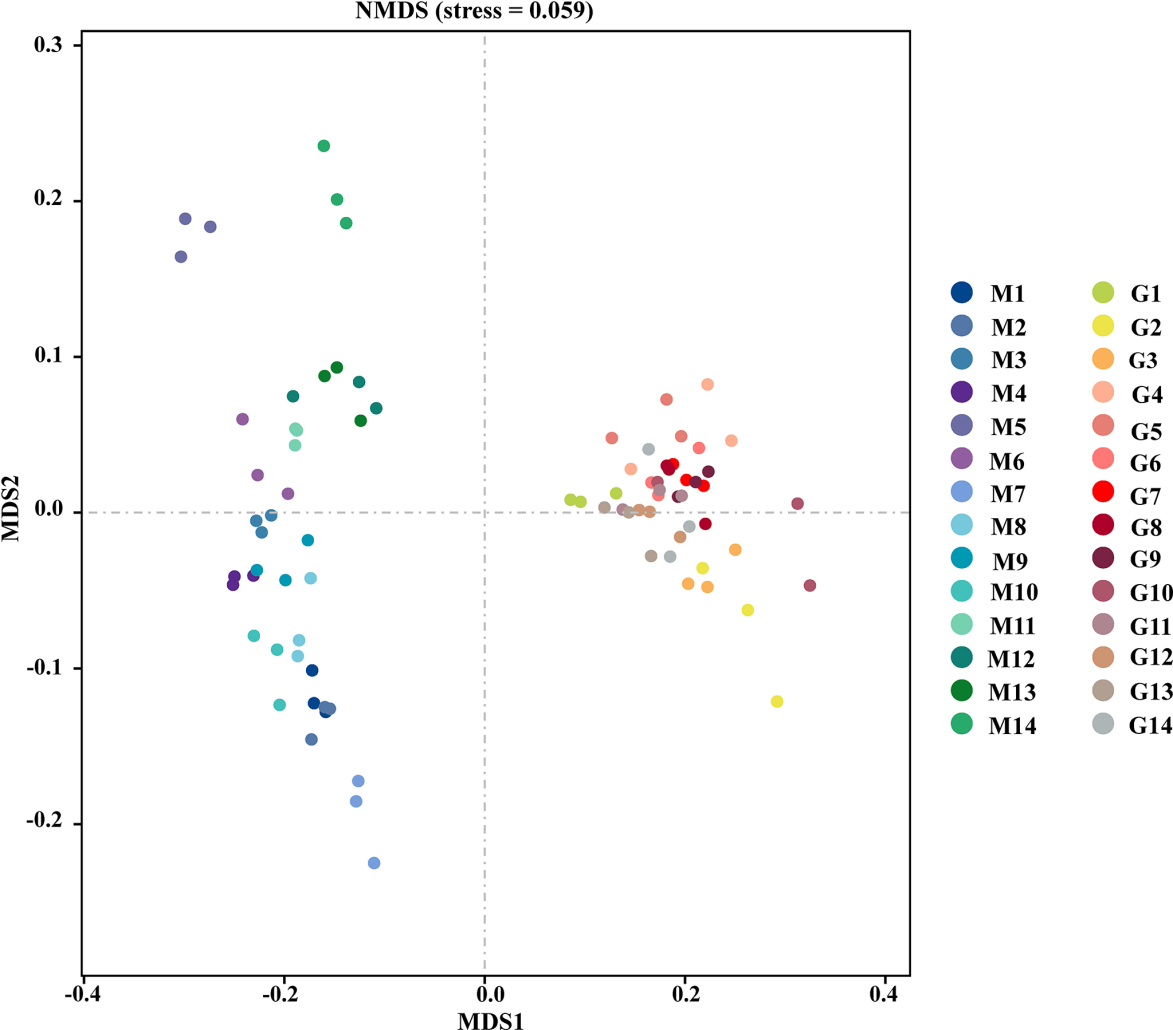
Unweighted unifrac nonmetric multidimensional scaling of the bacterial community compositions in the soil between group M and G.

The depth of sequencing in this experiment has basically covered all the species in the sample, as shown in the **Figure S1**. As shown in **Table 1**, the indices representing alpha-diversity were estimated as community richness and evenness. The diversity indices were lower in G compared with M, however, no significant differences in bacterial richness or diversity between M and G, as indicated by Sobs, Shannon, Simpson, Chao1, Pielou, and Pd. There are significant differences in coverage of ASV with low abundance between M and G, as indicated by Goods coverage index. The ACE index was significantly lower in G compared with M, showing that the richness and evenness of community in M were significantly higher than in G. NMDS was utillzed to analyze the comparability of the assemblage of bacterial communities between group M and G at the ASV level (**Figure 1**). Group M was located in the left part of the graph, and the distribution of different samples was discrete. The group G was gathered at the right, and the distribution of different samples was centralized. The results showed a distinctive difference in bacterial community structures between group M and G.

**Table 1:** Richness and alpha-diversity indices of the different ASVs between group M and G.

Group	Sobs			Shannon			Simpson			Chao1		
	Mean		SD	Mean		SD	Mean		SD	Mean		SD
**M**	4082.881a		379.9242	9.30265a		0.778844	0.98772a		0.020017	4951.628a		391.3516
**G**	3837.619a		357.408	9.00981a		0.748578	0.98145a		0.019449	4667.415a		368.1226
**Group**	**Ace**			**Goods coverage**			**Pielou**			**Pd**		
	**Mean**	**SD**		**Mean**	**SD**		**Mean**	**SD**		**Mean**	**SD**	
**M**	5125.335a	394.3544		0.98369a	0.001361		0.7754a	0.05825		359.3468a	26.01045	
**G**	4777.339b	366.216		0.98609b	0.001127		0.7562a	0.05588		340.168a	24.19418	

Different letters in the same column indicate a significant difference (ANOVA followed by Tukey s-b(k), n=14, p<0.05, average value, SD standard deviation).

### Taxonomic composition analysis

**Figure 2:**
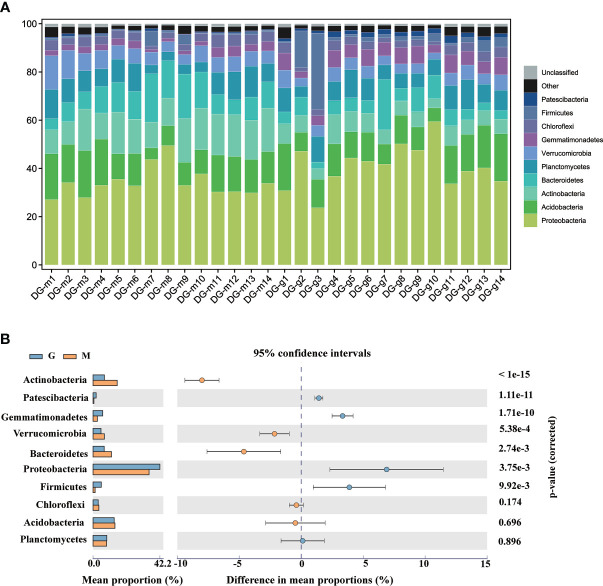
Taxonomic composition and abundance distribution of bacteria at the phylum level between group M and G **(A)**. The top 10 phyla with significant differences between group M and G **(B)**.

The species composition of rhizosphere microorganisms was analyzed at phylum and genus level. **Figure 2A** shows Proteobacteria, Acidobacteria, Actinobacteria, Bacteroidetes, Planctomycetes, Verrucomicrobia, Gemmatimonadetes, Chloroflexi, Firmicutes, Patescibacteria are most abundant at the phylum level, representing about 96.6% of the microorganisms detected in the 84 soil samples. Compared with group M, the abundance of Proteobacteria, Gemmatimonadetes, Patescibacteria, and Firmicutes have significantly (p <0.05) increased while the abundance of Actinobacteria, Bacteroidetes, Verrucomicrobia has significantly (p <0.05) decreased in group G. Proteobacteria, Acidobacteria, Actinobacteria were the most three abundant in the rhizosphere of *A. sinensis*. The relative abundance of Actinobacteria declined, showed a negative response to plant growth. The relative abundance of Proteobacteria increased, showed a positive response to plant growth. The relative abundance of Acidobacteria showed no significant difference during the plant growth. This observation is consistent with many other successional studies: including *Arabidopsis thaliana* (Roller et al., 2016), wheat (Bulgarelli et al., 2015), rice (Lu et al., 2006), switchgrass (Mao et al., 2014), maize (Peiffer et al., 2013) and *Avena barbata* (Zhalnina et al., 2018). It is indicating that the reconstruction of rhizosphere microbial communities had a general rule in different plant species and soil types. Research showed (Zhalnina et al., 2018), rhizosphere microbes that respond positively to plant growth are predicted to have longer generation times based on codon-usage bias, which means that their genomes are characterized by slower growth rates. Since slower-growing organisms can have higher substrate utilization efficiency (Chaparro et al., 2013), growth efficiency may be preferred over growth rate in the rhizosphere. Top 10 phyla with significant differences between group M and G are shown in **Figure 2B**.

**Figure 3:**
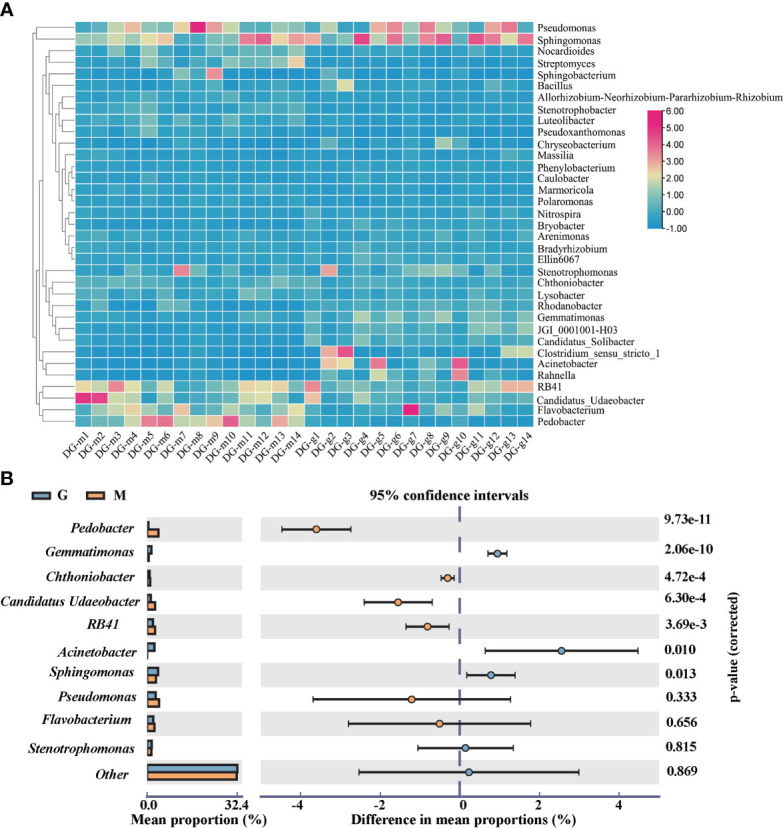
Heatmap of TOP 35 bacteria at the genus level between group M and G **(A)**. The top 10 genus with significant differences between group M and G **(B)**.

At the genus level, the dominant genera were *Pseudomonas, Sphingomonas, RB41, Flavobacterium, Pedobacter, Candidatus Udaeobacter, Stenotrophomonas, Acinetobacter, Gemmatimonas, Chthoniobacter*. The hierarchical clustering of TOP 35 genera showed that there were significant differences in microbial community structure between group M and G as shown in **Figure 3A**. The dominant genera also showed significant differences. Compared with group M, the abundance of *Acinetobacter, Gemmatimonas*, and *Sphingomonas* has significantly (p <0.05) increased while the abundance of *RB41, Pedobacter*, *Candidatus Udaeobacter, Chthoniobacter* has significantly (p <0.05) decreased in group G as shown in **Figure 3B**.

### Variations in metabolites of *Angelica sinensis* between M and G

**Figure 4:**
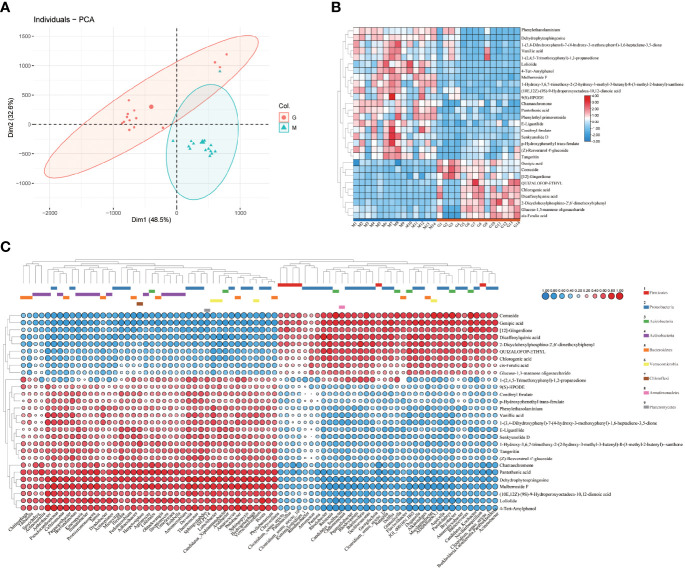
PCA scores for the comparison of metabolomic profiles between M and G **(A)**. Hierarchical clustering heatmap of the differential metabolites of *A. sinensis* between group M and G **(B)**. Covariation between differential abundant microbes and metabolites match against standards between group M and G (Spearman’s rank correlation) (n=3) **(C)**.

Untargeted metabolomics facilitates botanical metabolomics studies through efficient high-throughput screening techniques. At present, LC-MS/MS has been widely used for metabolomics studying (Tsugawa et al., 2021). In this study, UPLC-QTOF-MS/MS method was used to characterize metabolites in *A. sinensis* samples between M and G. Principal components analysis (PCA) (**Figure 4A**) and the OPLS-DA model (**Figure S2**) showed that the metabolic profiles varied significantly (*p* < 0.05) between groups, and the samples were clustered into two groups significantly. The hierarchical clustering heatmap (**Figure 4B**) showed the differences between group M and G, suggesting that the synthesis of secondary metabolites pattern at seedling stage and collection period of medicinal material are distinct. Further analysis (**Table S1**) combined with a hierarchical clustering heatmap (**Figure 4B**) revealed that group G had a higher level of organic acids, such as dicaffeoylquinic acid, chlorogenic acid, genipic acid, and ferulic acid. In addition, the contents of comuside, phenylethyl primeveroside and QUIZALOFOP-ETHYL, 2-dicyclohexylphosphino-2’,6’-dimethoxybiphenyl and [12]-gingerdione in group G were higher than M, while the levels of phenylpropanoids, phthalide and other organoheterocyclic compounds, flavonoids, amines, and fatty acid were lower compared to group M. These results suggest that different growth stages of *A. sinensis* have different secondary metabolite synthesis patterns, which indicate that changes in the composition of the secondary metabolites over time may contribute to the observed successional patterns in the rhizosphere microbiota (**Figure 1**). Metabolites with the Variable Importance for Projection (VIP) value greater than 1 and the p-value less than 0.05, combined with s-plot (covariance greater than 0.05) were screened as markers that contributing to grouping. A total of 29 markers were identified. (**Table 2**) (Zhang et al., 2019).

**Table 2:** Differential metabolites for discriminating *A. sinensis* samples between M and G.

Identification	tR(min)	Theoretical accurate mass (m/z)	Q-TOF-MS (m/z)(ESI+/ESI-)	Mass accuracy (ppm)	MS/MS fragment ion (m/z)
Glucose-1,3-mannose oligosaccharide	1.05	341.1089[M-H]-	341.1072[M-H]-	-4.98	
Phenylethanolaminium	1.53	120.0814[M+H-H2O]+	120.0811[M+H-H2O]+	-2.50	
Pantothenic acid	1.72	218.1034[M-H]-	218.1016[M-H]-	-8.25	
Vanillic acid	3.66	167.0344[M-H]-	167.0334[M-H]-	-5.99	123.0447[M-H]-
Mulberroside F	3.85	401.1453[M-H]-	401.1439[M-H]-	-3.49	565.1557[M-H]-、387.1641[M-H]-
Chamaechromone	3.99	541.1140[M-H]-	541.1182[M-H]-	7.76	
Phenylethyl primeveroside	4.79	415.1610[M-H]-	415.1592[M-H]-	-4.34	
cis-Ferulic acid	5.19	177.0552[M+H-H2O]+	177.0553[M+H-H2O]+	0.56	
Chlorogenic acid	5.99	353.0873[M-H]-	353.0863[M-H]-	-2.83	
Dicaffeoylquinic acid	6.32	515.119[M-H]-	515.1181[M-H]-	-1.75	
QUIZALOFOP-ETHYL	6.4	353.0693[M-H20-H]-	353.0701[M-H20-H]-	2.27	
(Z)-Resveratrol 4’-glucoside	6.7	389.1242[M-H]-	389.1223[M-H]-	-4.88	373.1855[M-H]-
Tangeritin	8.22	371.1136[M-H]-	371.1119[M-H]-	-4.58	
Loliolide	8.7	177.0916[M-H2O-H]-	177.0903[M-H2O-H]-	-7.34	
4-Tert-Amylphenol	10.15	163.1128[M-H]-	163.1113[M-H]-	-9.20	
1-(2,4,5-Trimethoxyphenyl)-1,2-propanedione	10.48	219.0657[M-H20-H]-	219.0645[M-H20-H]-	-5.48	
p-Hydroxyphenethyl trans-ferulate	10.64	313.1076[M-H]-	313.1064[M-H]-	-3.83	134.0361[M-H]-、193.0495[M-H]-
1-(3,4-Dihydroxyphenyl)-7-(4-hydroxy-3-methoxyphenyl)-1,6-heptadiene-3,5-dione	11.34	353.1031[M-H]-	353.1016[M-H]-	-4.25	203.0697[M-H]-
Coniferyl ferulate	11.34	353.1031[M-H]-	353.1016[M-H]-	-4.25	193.0488[M-H]-、134.0362[M-H]-
E-Ligustilide	14.55	191.1072[M+H]+	191.1069[M+H]+	-1.57	
Comuside	15.46	313.1156[M+2ACN+2H]+	313.1154[M+2ACN+2H]+	-0.64	
Senkyunolide D	15.48	221.0814[M-H]-	221.0804[M-H]-	-4.52	
Dehydrophytosphingosine	15.87	316.2846[M+H]+	316.2853[M+H]+	2.21	
(10E,12Z)-(9S)-9-Hydroperoxyoctadeca-10,12-dienoic acid	15.88	311.2228[M-H]-	311.221[M-H]-	-5.78	
9(S)-HPODE	20	293.2117[M-H20-H]-	293.2104[M-H20-H]-	-4.43	
Genipic acid	20.42	185.0808[M+H]+	185.0816[M+H]+	4.32	
2-Dicyclohexylphosphino-2’,6’-dimethoxybiphenyl	22	409.2302[M-H]-	409.2344[M-H]-	10.26	
1-Hydroxy-3,6,7-trimethoxy-2-(2-hydroxy-3-methyl-3-butenyl)-8-(3-methyl-2-butenyl)-xanthone	23.15	453.1919[M-H]-	453.1938[M-H]-	4.19	
[12]-Gingerdione	23.5	377.2686[M+H]+	377.2673[M+H]+	-3.45	

### Quality evaluation in medicinal components of *Angelica sinensis* between M and G

**Figure 5:**
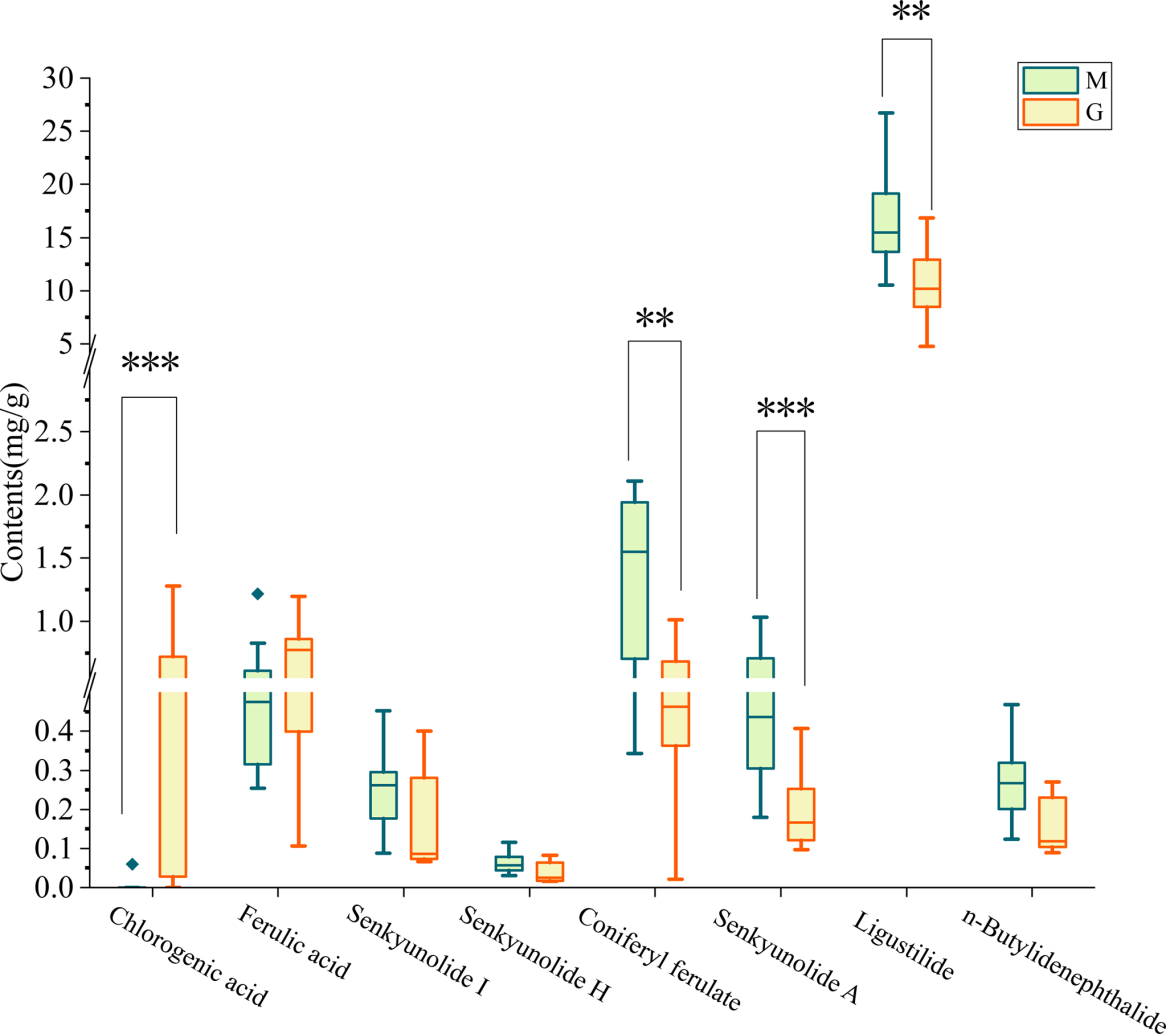
Comparison of the medicinal components of *A. sinensis* samples between group M and G. **P ≤ 0.01; ***P ≤0.001.

To further explain the cause of differences in metabolites of *A. sinensis* between group M and G, phthalides and organic acids were comprehensively analyzed. The result showed that the contents of medicinal components differed remarkably (p < 0.05) between groups. Specially, the level of coniferyl ferulate, senkyunolide A, and ligustilide of *A. sinensis* in group M were significantly (p < 0.01) higher than in group G (**Figure 5**). The level of chlorogenic acid in group G was significantly higher than in group M (p < 0.001).

### The characteristics of metabolites and rhizosphere microbial communities succession of *Angelica sinensis* over growth

The utilization of specific chemical components of exudates by microorganisms has potential metabolic differences, which may be crucial for bacterial success in the rhizosphere (Zhalnina et al., 2018). Thus, the chemical composition of plant secondary metabolites synthesis and plant exudates may represent key means of shaping the microbial composition of the rhizosphere. The hierarchical clustering heatmap showed that the differential metabolites were clustered into two groups (**Figure 4C**), and the distribution and type of differential metabolites are highly consistent with **Figure 4B**. The results showed that the rhizosphere microbial community have different assembly patterns between group M and G, and the rhizosphere microbial community had a key contribution to grouping and quality-related factors of *A. sinensis.* Combined **Figures 4B, C**, we found that Actinobacteria, Proteobacteria and Bacteroidetes were predominant in synthesizing secondary metabolites such as phenylpropanoids, phthalide, and other organoheterocyclic compounds, flavonoids, amines, and fatty acid in the seedling stage or early growth stage of *A. sinensis*. Proteobacteria, Acidobacteria and Firmicutes were predominant in the synthesis of organic acid in the drug-producing stage or later growth stage of *A. sinensis*. Aromatic organic acids, such as cinnamic acid, have been found to shape plant rhizosphere microbes and influence plant-microbe interactions (Sasse et al., 2018; Cotton et al., 2019). Therefore, the synthesis of medicinal components and differential secondary metabolites is closely related to the growth stage of *A. sinensis*. The synthesis and secretion of secondary metabolites also affect the assembly of the rhizosphere microbial community.

### Correlation between the differential microbial community, differential secondary metabolites and medicinal components

**Figure 6:**
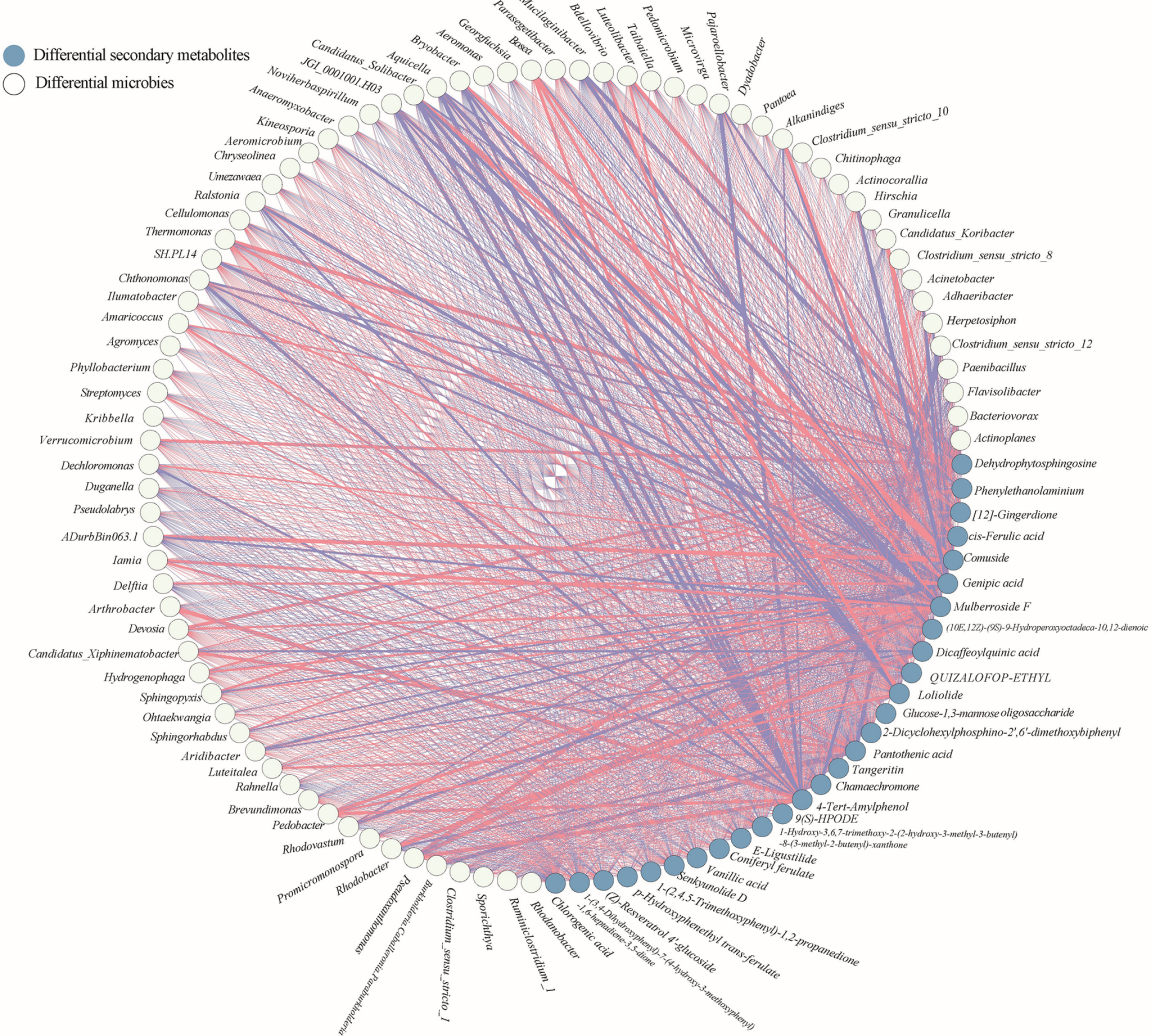
Co-occurrence network of the differential secondary metabolites and differential microbial taxa in rhizosphere of *A. sinensis*.

**Figure 7:**
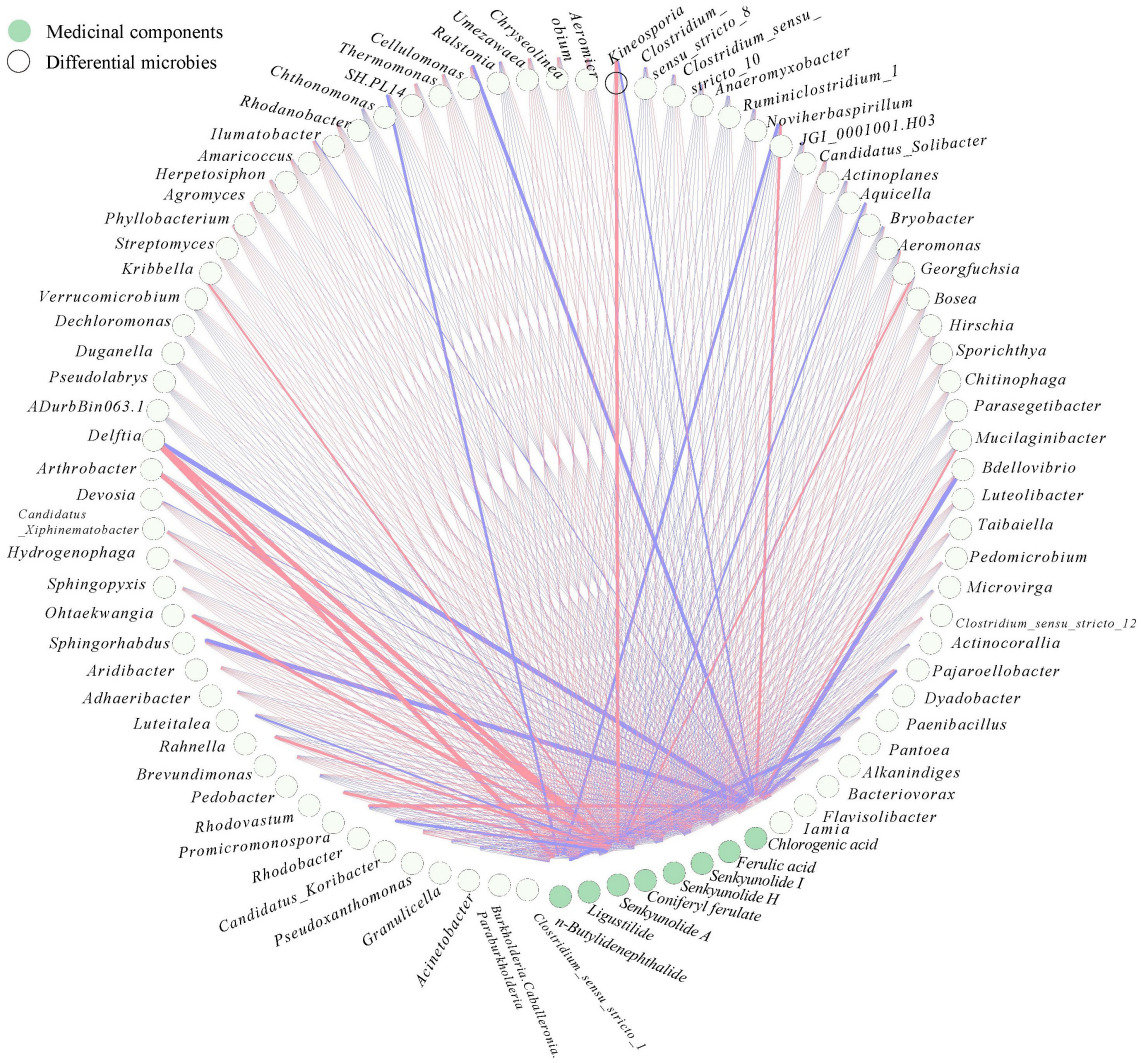
Co-occurrence network of the medicinal components and differential microbial taxa in rhizosphere of *A. sinensis*.

In the case pf medicinal plants, specific microorganisms may be directly related to the biosynthesis of medicinal components of the host plant. For example, *Lysobacter* was identified strongly associated with gene CYP72A154, which was required glycyrrhizic acid biosynthesis of *Glycyrrhiza uralensis Fish* (Zhong et al., 2022). Therefore, to further elucidate the relation between differential microbial community, differential secondary metabolites and medicinal components, two interactive networks were constructed (**Figures 6**, **7**). The significance test and correlation of differential microbial community, differential secondary metabolites and medicinal components were showed in **Figures S3**, **S4**.

### Correlation between differential microbial community and differential secondary metabolites

**Figure 8:**
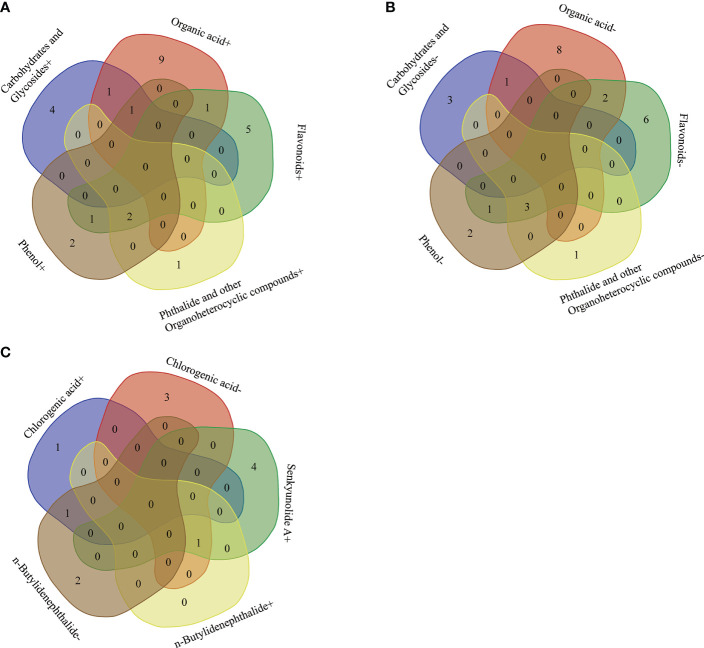
Venn comparative analysis of main differential secondary metabolites of *A*. *sinensis* and differential microbes correlated positively **(A)**. Venn comparative analysis of main differential secondary metabolites of *A*. *sinensis* and differential microbes correlated negatively **(B)**. Venn comparative analysis of the correlation between differential medicinal components of *A*. *sinensis* and differential microbes **(C)**.

There showed a complex correlation between differential microbial community and differential secondary metabolites (**Figure 6**). For differential secondary metabolites, the flavonoids mulberroside F showed the greatest correlation with the differential bacterial members (**Figure 6**), and the phenol 4-Tert-Amylphenol, the amines dehydrophytosphingosine, the organic acid genipic acid and pantothenic acid, the organoheterocyclic loliolide are also important nodes that correlate with the differential bacterial members. 4-Tert-Amylphenol, loliolide and mulberroside F were negatively correlated with *JGI 0001001.H03, Candidatus Solibacter, Aquicella* and *Bryobacter*, while genipic acid and comuside was positively correlated with *Candidatus Solibacter* and *Bryobacter*, genipic acid was positively correlated with *Candidatus Solibacter* (r > 0.7). Various rhizodeposits may differentially influence the composition of the rhizosphere microbiome composition (Pascale et al., 2020). Recently studies showed that selected secondary including flavonoid, coumarin, benzoxazinoid, phytohormones, and triterpenes affect the succession of rhizosphere microorganisms of host plants. (Pang et al., 2021). In this paper, we classified the differential secondary metabolites of *A. sinensis* according to its chemical structure, and made a further Venn comparative analysis on the primary, and secondary metabolites of carbohydrates and glycosides, organic acid, flavonoids, phthalides, and other organoheterocyclic compounds, phenol (**Figures 8A, B**). The results showed that carbohydrates and glycosides, organic acid, and phenol were positively correlated with *Candidatus Koribacter* (r > 0.7), carbohydrates and glycosides, organic acid were positively correlated with *Candidatus Solibacter* (r > 0.7), flavonoids, phenol, Phthalide, and other Organoheterocyclic compounds were positively correlated with *Pedobacter* and *Bosea* (r > 0.7), flavonoids and organic acid were positively correlated with *Iamia* (r > 0.7), flavonoids and phenol were positively correlated with *Thermomonas* (r > 0.7).

Flavonoids, phenol, phthalide, and other organoheterocyclic compounds were negatively correlated with *JGI 0001001.H03, Candidatus Solibacter, Aquicella* (r > 0.7), carbohydrates and glycosides, organic acids were negatively correlated with *Clostridium sensu stricto 12*, flavonoids and organic acid were negatively correlated with *Burkholderia.Caballeronia.Paraburkholderia* (r > 0.7), flavonoids and phenol were negatively correlated with *Ralstonia* (r > 0.7). From the above results, we can see that flavonoids, phthalides, and other organoheterocyclic compounds have consistent correlation with specific bacterial communities; carbohydrates and Glycosides, and organic acids have consistent correlation with the other specific bacterial communities, which have opposite trend with the former. In short, flavonoids, organic acids, carbohydrates and glycosides, phthalide, and other organoheterocyclic compounds are strongly related to microbes, which may be the main driving factors of microbial community assembly in the rhizosphere of *A. sinensis*.

### Correlation between the medicinal compounds and differential bacterial community

There showed a complex correlation between differential microbial community and medicinal components (**Figure 7**). Chlorogenic acid, senkyunolide A, and n-butylidenephthalide showed the greatest correlation with rhizosphere bacterial communities among 8 medicinal components. Chlorogenic acid was positively correlated with *Rhodobacter, JGI 0001001.H03* (r > 0.7), while negatively correlated with *Aridibacter, Arthrobacter, Cellulomonas, Luteolibacter* (r > 0.7). Senkyunolide A was positively *Kineosporia, Arthrobacter, Devosia, Sphingorhabdus, and Brevundimonas* (r > 0.7). n-butylidenephthalide was positively with *Arthrobacter*, while negatively correlated with *Alkanindiges, JGI 0001001.H03, Chthonomonas* (r > 0.7). Interestingly, the Venn comparative analysis showed that *Arthrobacter* was positively correlated with senkyunolide A and n-butylidenephthalide, while negatively correlated with chlorogenic acid (r > 0.7) (**Figure 8C**). And *JGI 0001001.H03* was positively correlated with chlorogenic acid, while negatively correlated with n-butylidenephthalide (r > 0.7) (**Figure 8C**). In the previous analysis, the level of chlorogenic acid in group G was significantly higher than in group M (p < 0.001), while the level of senkyunolide A was significantly lower than in group M (p < 0.001). And the content of n-butylidenephthalide in group M was also higher than in group G. Therefore, *Arthrobacter* and *JGI 0001001.H03* may be the key microorganism related to the quality of *A. sinensis*. *Arthrobacter* is known for its nutritionally versatile nature and wade prevalence in stressful environments, with efficient survivability under high attitude stress conditions (Mukhia et al., 2021). And the strains of *Arthrobacter* also have the ability to degrade Dibutyl phthalate (DBP), dimethyl phthalate (DMP), 4-chlorophenol efficiently (Kim et al., 2008; Wang et al., 2019; Liu et al., 2020). Therefore, we speculate that *Arthrobacter* may be able to provide energy to *A. sinensis* decomposing harmful substances in high altitude and cold environment, and promote the synthesis of medicinal components such as n-butylidenephthalide and senkyunolide A in the early growth of *A. sinensis*.

## Discussion

The primary active components of *A. sinensis* include phthalides, organic acids and polysaccharides (Wei et al., 2016). Research on the production of medicinal bioactive components from rhizosphere microbiome and metabolomic perspective for medicinal plants is still lacking. Since phthalides and organic acids are the marker components for assessing the quality of *A. sinensis*, studying how to stimulate the accumulation of these two main bioactive components has become a key technical point for improving the quality of *A. sinensis*. The accumulation of secondary metabolite and the rhizosphere microbes are essential for improved active constituents of *A. sinensis*. Here, we measured root metabolites, contents of medicinal components, and sequenced the rhizosphere microbes for *A. sinensis* under seedling stage and picking stage. We exploited quality-related mechanisms of authentic *Angelica sinensis* using a combination of metabolites content and microbial community to identify regulation of microbes and secondary metabolites synthesis mechanisms.

Extensive studies have proved the soil microenvironment influenced the growth and level/type of active components in *A. sinensis* (Zhu et al., 2021). In this study, we found that the developmental stage is also an important factor affecting the accumulation of medicinal components of *A. sinensis*. Compared with seedling stage of *A. sinensis*, the level of chlorogenic acid in picking stage was significantly increased, while the level of coniferyl ferulate, senkyunolide A and ligustilide were significantly decreased in picking stage (**Figure 5**). It has been shown that the accumulation of secondary metabolites of *A. sinensis* is tightly connected to the growth periods. The four chemical markers of *A. sinensis* are Z-ligustilide, Z-butylidenephalide, butylphthalide, linoleic acid, which were remarkable differences in their contents during growth, and concentrations of these markers were relatively higher in September and October (Qian et al., 2013). Through further analysis of metabolomics of *A. sinensis* root, we found that secondary metabolites had significant differences between different growth stages (seedling stage and picking stage) of *A. sinensis*. Organic acid, such as dicaffeoylquinic acid, chlorogenic acid, genipic acid, and ferulic acid, were mostly synthesized at the picking stage. While the levels of phenylpropanoids, phthalide, and other organoheterocyclic compounds, flavonoids, amines, and fatty acids were lower compared to seedling stage, indicating that different growth stages of *A. sinensis* have different secondary metabolite synthesis patterns. Root exudates are a mix of a wide variety of compounds, including primary and secondary metabolites. Primary metabolites, including carbohydrates, amino acids, and organic acids, are secreted in larger quantities than secondary metabolites, such as flavonoids, glucosinolates, auxins.etc. Among them, the differential metabolites of group M and G, such as ferulic acid, vanillic acid, pathothenic and glucosides can be secreted to the rhizosphere (Badri et al., 2009; Vives-Peris et al., 2020). The composition of root exudates is not constant, its composition varies with developmental stage, environmental conditions, plant species, soil type, nutrition, and root traits, and among other factors. (Jones, 1998; Aulakh et al., 2001; Badri and Vivanco, 2009; Iannucci et al., 2013). In maize, several studies have shown that benzoxazinoids (BXs) are synthesized and secreted in plant early stage that display allelopathic activities and insecticidal action, and affect root -related microbiota by inhibiting plant pathogens and colonization by specific microbial taxa (Hu et al., 2018; Kudjordjie et al., 2019). Released compounds have been shown to influence the assembly of rhizosphere microbiota, thus improving the ability of plants to adapt to their environments (Bulgarelli et al., 2013).

Correlation between differential secondary metabolites and differential microbes were linked to the quality of *A. sinensis*, as well as environmental factors. Our study revealed a regulatory microbe-metabolite network for the correlation of microorganisms and differential metabolites at the molecular level, showed that the composition of rhizosphere microbes in picking stage were dominated by proteobacteria, which had a strong correlation with the synthesis of organic acids. The composition of rhizosphere microbes in seedling stage were dominated by Actinobacteria, which had a strong correlation with the synthesis of phthalide and other organoheterocyclic compounds, flavonoids, amines, and fatty acid. On the one hand, the succession of rhizosphere microbial communities based on different growth stages is related to bacteria generation time, as slower-growing microorganisms have higher rhizodeposits utilization efficiency (Roller et al., 2016). On the other hand, the growth and succession of bacteria in the rhizosphere is not only determined by the root exudates, but also can be predicted by the substrate preferences of rhizosphere bacteria. This study provides direct evidence that specific rhizosphere exudates manipulate rhizosphere microbial community assembly. In the rhizosphere, substrate preferences may provide a selection advantage (Zhalnina et al., 2018). Proteobacteria contained significantly more organic acid transporter genes than Actinobacteria, suggesting that positive and negative bacteria respond to plant growth differed in their predicted metabolic potential to utilize organic acids (Zhalnina et al., 2018). These factors interact and affect the assembly patterns of the rhizosphere microbial community of *A. sinensis* at different growth stages. Therefore, we speculate that the stage-specific microbes may be associated with the particular root exudates that drive the microbes to respond quickly (Zhang et al., 2017).

## Conclusion

In this study, we have explored quality-related mechanisms of authentic *A. sinensis* at the metabolite and microbiota levels based on samples, and first identified the differences of *A. sinensis* under different growth status at the medicinal components and metabolite levels: we have confirmed that *A. sinensis* at the picking stage accumulated significantly more chlorogenic acid than seedling stage, while the contents of coniferyl ferulate, senkyunolide A and ligustilide were significantly decreased. The synthesis of differential secondary metabolites at different growth stages also showed a similar trend: organic acids were mainly synthesized at the picking stage, while phenylpropanoids, phthalide, and other organoheterocyclic compounds, flavonoids, amines, fatty acids were mainly synthesized at the seedling stage. The differences in the biosynthesis of different types of secondary metabolites over growth are also related to the changes of composition of microorganisms in the rhizosphere of *A. sinensis*. Our study showed that the composition of rhizosphere microbes in picking stage were dominated by proteobacteria, which had a strong correlation with the synthesis of organic acids. The composition of rhizosphere microbes in seedling stage were dominated by Actinobacteria, which had a strong correlation with the synthesis of phthalide and other organoheterocyclic compounds, flavonoids, amines, and fatty acid. Secondly, in view of the co-occurrence network analysis, we have comprehension of the integrated microbe-medicinal associations. This finding was exemplified by rhizosphere microbes *Arthrobacter* and *JGI 0001001.H03*, which were thought to be the key microorganisms related to the quality of *A. sinensis*. *Arthrobacter* was found to be strongly associated with the accumulation of senkyunolide A and n-butylidenephthalide, *JGI 0001001.H03* was found to be strongly associated with the accumulation of chlorogenic acid. Microbe-differential secondary metabolites indicated that flavonoids, organic acids, carbohydrates and glycosides, phthalide, and other organoheterocyclic compounds were closely and strongly related to differential rhizosphere microbes, which may be the main driving factors affecting the assemble of microbial community in rhizosphere of *A. sinensis*. Collectively, These findings provide such a basis for further exploration into the relationship between rhizosphere microorganisms and the medicinal bioactive markers over growth, are also helpful in guiding future cultivation of *A. sinensis.*

## Data availability statement

The datasets presented in this study can be found in online repositories. The names of the repository/repositories and accession number(s) can be found at: https://www.ncbi.nlm.nih.gov/, PRJNA893538.

## Author contributions

The experiments were conceived and designed by HY, PL, and J-AD. The experiments were carried out by J-MC, who also drafted the manuscript. J-MC and W-MF analyzed the data. HY, W-MF, PL also contributed to manuscript editing, literature search, and figures generation. G-SZ, SG, GY, and J-AD contributed to the discussion of the manuscript. All authors contributed to the article and approved the submitted version.

## Funding

This research was supported financially by National Natural Science Foundation of China (81773848), Innovation Team and Talents Cultivation Program of National Administration of Traditional Chinese Medicine (ZYYCXTD-D-202005), China Agriculture Research System of MOF and MARA (CARS-21), Ministry of Finance Central Level of the Special (2060302). This work was also partly sponsored by Jiangsu Province 333 Highlevel Talents Training Project, Qing Lan Project, Six talents peaks project in Jiangsu Province (JNHB-066).

## Acknowledgments

Thanks to Guangzhou Genedenovo Biotechnology Co., Ltd. for helping with the sequencing and bioinformatics analysis. We also thank Zeng-Xiang Guo for assisting with the seedling samples, technical support for field management, and permission to conduct research at Min County *Angelica sinensis* institute.

## Conflict of interest

The authors declare that the research was conducted in the absence of any commercial or financial relationships that could be construed as a potential conflict of interest.

## Publisher’s note

All claims expressed in this article are solely those of the authors and do not necessarily represent those of their affiliated organizations, or those of the publisher, the editors and the reviewers. Any product that may be evaluated in this article, or claim that may be made by its manufacturer, is not guaranteed or endorsed by the publisher.

## Supplementary material

Supplementary Figure 1 Species accumulation curve of bacteria.

Supplementary Figure 2 OPLS-DA for the comparison of metabolomic profiles between M and G.

Supplementary Figure 3 Correlation between the differential secondary metabolites and differential microbial taxa in rhizosphere of *A. sinensis.* (*, *P* <=0.05; **, *P* <= 0.01; ***, *P* <=0.001.).

Supplementary Figure 4 Correlation between the medicinal components and differential microbial taxa in rhizosphere of *A. sinensis.* (*, *P* <=0.05; **, *P* <= 0.01; ***, *P* <=0.001.).

## Abbreviations

PSCs, Plant secondary compounds; UPLC-QTOF-MS/MS, Ultra Performance Liquid Chromatography-Quadrupole Times of Flight-Mass/Mass; LC-MS/MS, liquid chromatography coupled with high-resolution tandem MS; PCA, Principal components analysis; OPLS-DA, Orthogonal partial least-squares discrimination analysis; NMDS, Nonmetric multidimensional scaling; VIP, Variable importance for projection; ANOVA, Analysis of varia; OUT, Operational taxonomic units; PCR, Polymerase chain reaction; DBP, Dibutyl phthalate; DMP, Dimethyl phthalate; BXs, Benzoxazinoids.
