# Supplementary figures and images for: Explore the interaction between root metabolism and rhizosphere microbiota during the growth of Angelica sinensis
Source: Front Plant Sci. 2022 Nov 7;13:1005711. doi: 10.3389/fpls.2022.1005711 (PMC9676459; doi:10.3389/fpls.2022.1005711)

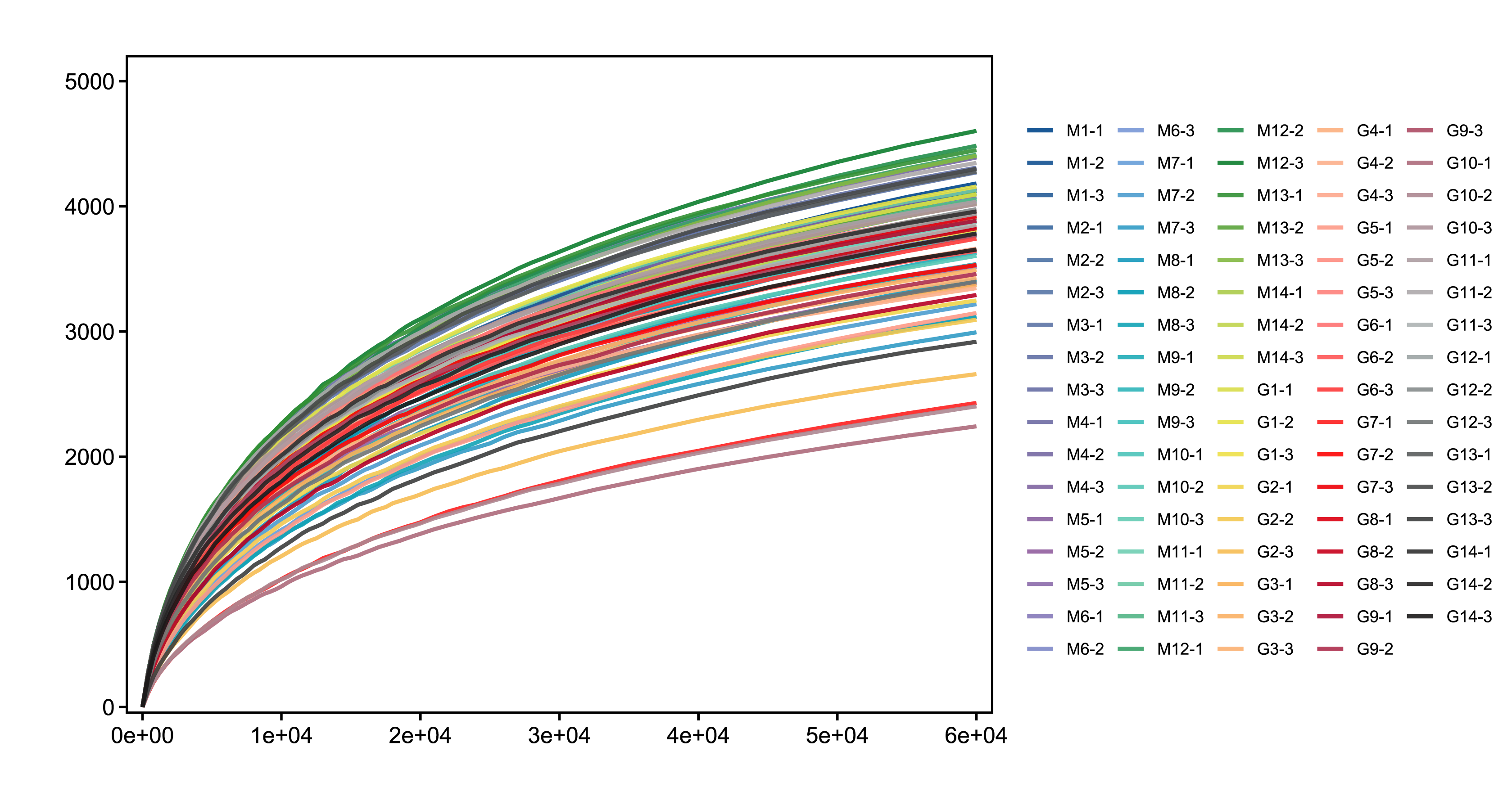

Supplement: Supplementary Figure 1 — Species accumulation curve of bacteria. [file Image_1.png]

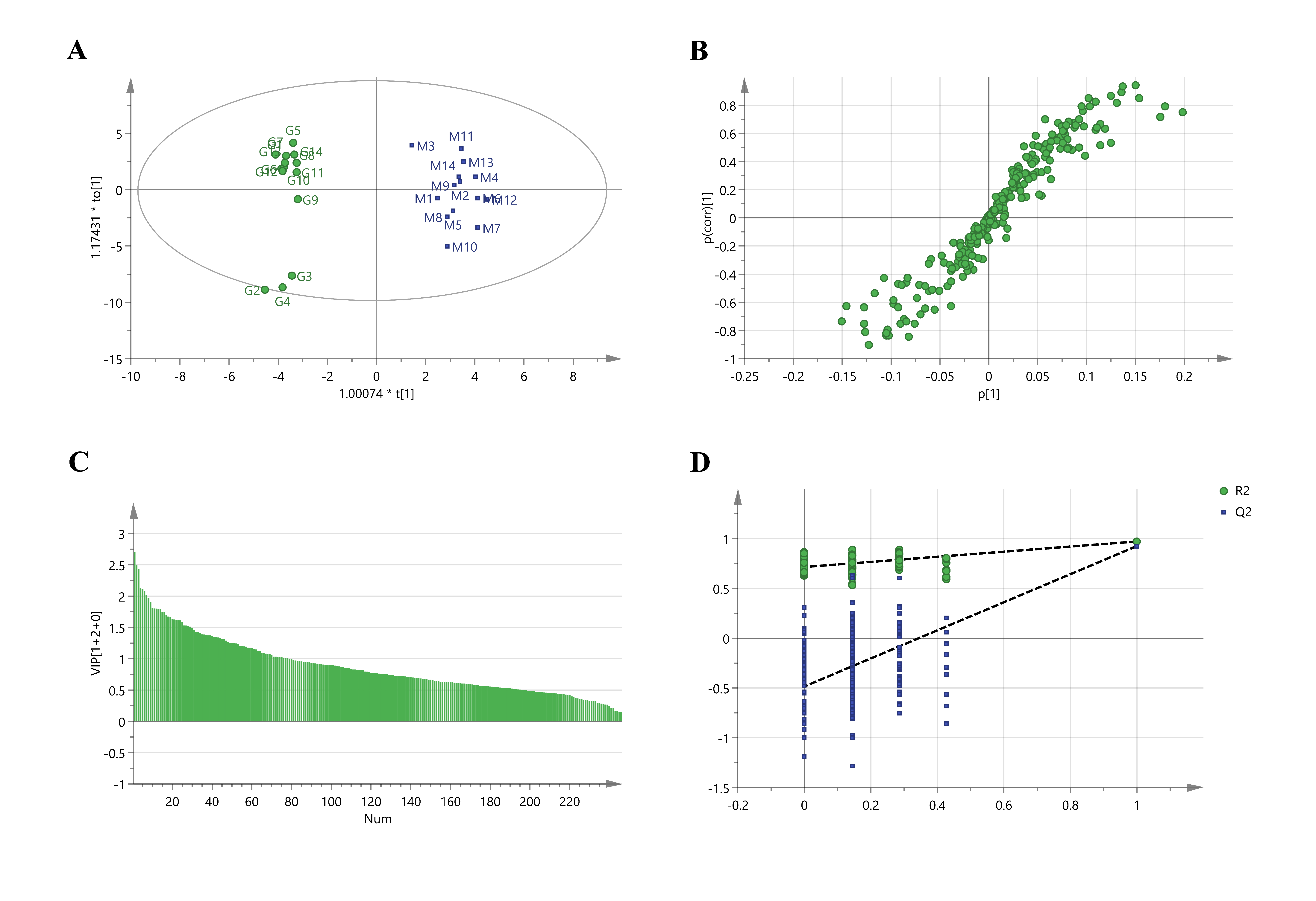

Supplement: Supplementary Figure 2 — OPLS-DA for the comparison of metabolomic profiles between M and G. [file Image_2.tif]

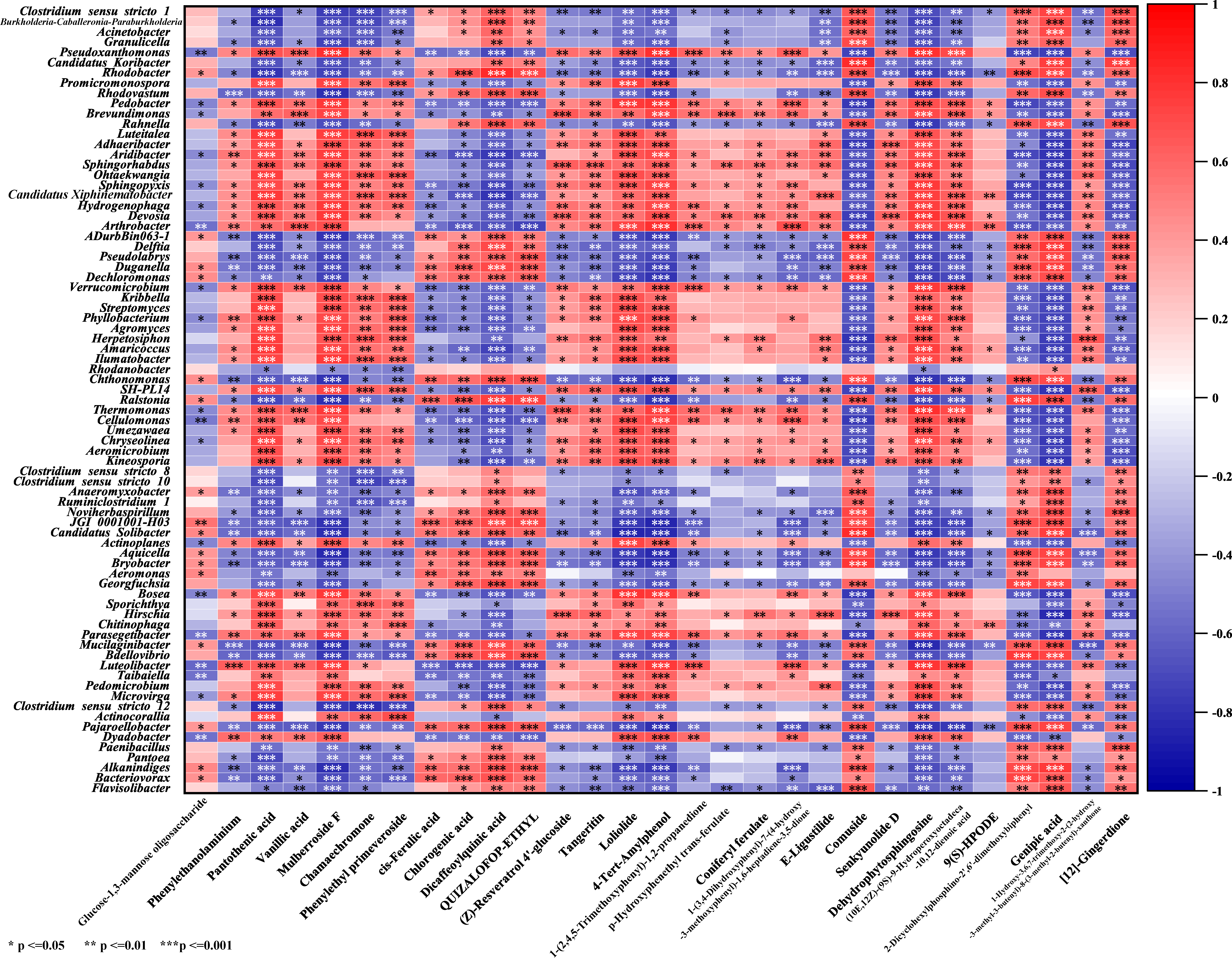

Supplement: Supplementary Figure 3 — Correlation between the differential secondary metabolites and differential microbial taxa in rhizosphere of A. sinensis. (*, P <=0.05; **, P <= 0.01; ***, P <=0.001.). [file Image_3.tif]

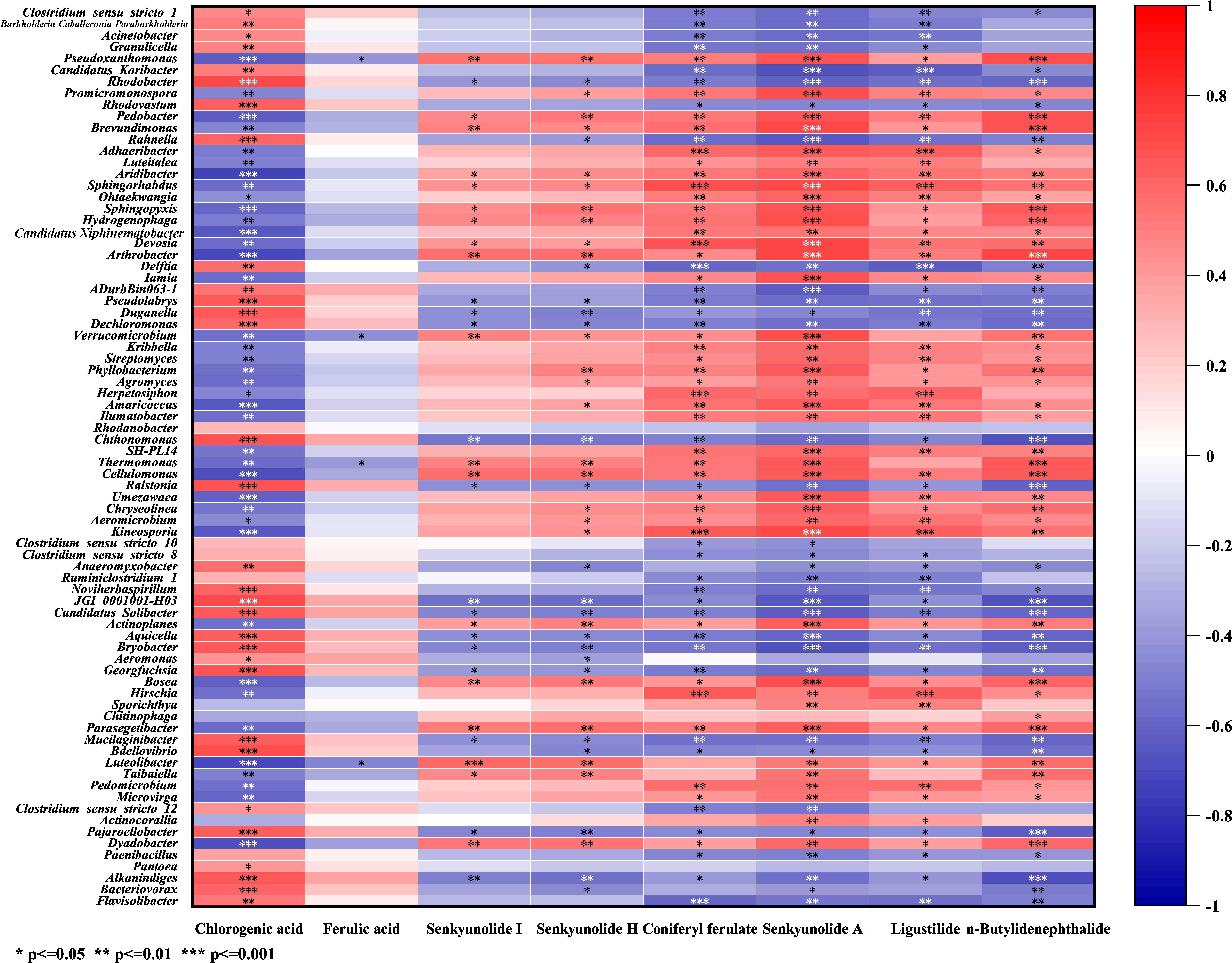

Supplement: Supplementary Figure 4 — Correlation between the medicinal components and differential microbial taxa in rhizosphere of A. sinensis. (*, P <=0.05; **, P <= 0.01; ***, P <=0.001.). [file Image_4.tif]
